# Tumour-associated endothelial-FAK correlated with molecular sub-type and prognostic factors in invasive breast cancer

**DOI:** 10.1186/1471-2407-14-237

**Published:** 2014-04-02

**Authors:** Annika N Alexopoulou, Colan M Ho-Yen, Vassilis Papalazarou, George Elia, J Louise Jones, Kairbaan Hodivala-Dilke

**Affiliations:** 1 Adhesion and Angiogenesis Laboratory, Centre for Tumour Biology, Barts Cancer Institute – a CR-UK Centre of Excellence, Queen Mary University of London, John Vane Science Centre, Charterhouse Square, London EC1M 6BQ, UK; 2 Breast Group, Centre for Tumour Biology, Barts Cancer Institute – a CR-UK Centre of Excellence, Queen Mary University of London, John Vane Science Centre, Charterhouse Square, London EC1M 6BQ, UK; 3 Vascular Adhesion Lab, BSRC Al. Fleming, 34 Fleming str., 166 72 Vari Athens, Greece

**Keywords:** FAK, Endothelium, Breast cancer, Molecular sub-type

## Abstract

**Background:**

Breast cancer is a heterogeneous disease that can be classified into one of 4 main molecular sub-types: luminal A, luminal B, Her2 over-expressing and basal-like (BL). These tumour sub-types require different treatments and have different risks of disease progression. BL cancers can be considered a sub-group of Triple negative (TN) cancers since they lack estrogen (ER), progesterone (PR) and Her2 expression. No targeted treatment currently exists for TN/BL cancers. Thus it is important to identify potential therapeutic targets and describe their relationship with established prognostic factors. Focal adhesion kinase (FAK) is upregulated in several human cancers and also plays a functional role in tumour angiogenesis. However, the association between breast cancer sub-types and tumour endothelial-FAK expression is unknown.

**Methods:**

Using immunofluorescence, we quantified FAK expression in tumour endothelial and tumour cell compartments in 149 invasive breast carcinomas and correlated expression with clinical, pathological and molecular parameters.

**Results:**

Low endothelial-FAK expression was independently associated with luminal A tumours at univariate (p < 0.001) and multivariate (p = 0.001) analysis. There was a positive correlation between FAK expression in the vascular and tumour cell compartments (Spearman’s correlation co-efficient = 0.394, p < 0.001). Additionally, endothelial and tumour cell FAK expression were significantly increased in TN tumours (p = 0.043 and p = 0.033 respectively), in tumours with negative ER and PR status, and in high grade tumours at univariate analysis.

**Conclusion:**

Our findings establish a relationship between endothelial-FAK expression levels and the molecular sub-type of invasive breast cancer, and suggest that endothelial-FAK expression is potentially more clinically relevant than tumour cell FAK expression in breast cancer.

## Background

Breast cancer is a heterogeneous disease which, according to extensive gene expression profiling, can be grouped into 4 major categories: luminal A, luminal B, human epidermal growth factor receptor-2 oncogene (also called Her2/ERBB2) type and basal-like breast cancer [1,2]. Each tumour type requires different treatment, has a different risk of disease progression and distinct patterns of metastasis [3]. Therefore, ER tumours are treated using anti-estrogen based therapies such as tamoxifen or aromatase inhibitors and Her2 over-expressing tumours can be targeted with the anti-Her2 therapy trastuzumab. The aggressive basal-like (BL) tumours can be considered a sub-group of triple negative (TN) tumours since most are negative for ER, PR and Her2 [4,5]. TN/BL cancers have a poor prognosis in comparison to other molecular sub-types and targeted molecular therapies are not currently available for patients with these tumours. Thus identifying new therapeutic targets becomes a priority for TN/BL cancers.

Focal adhesion kinase (FAK) is a 125 kDa non-receptor tyrosine kinase that can be activated both by integrins and extracellular stimuli such as growth factors [6,7]. FAK is involved in, and regulates, several key cell processes in cancer progression and tumour angiogenesis including cell survival and apoptosis, adhesion, migration and invasion.

In human cancers, increased tumour cell FAK expression has been shown in several cancer types including lung, cervical, colon and breast when compared to normal tissue [8-12]. In non-small–cell lung cancer high tumour cell FAK expression was found to correlate with increased lymph node metastasis and decreased survival [8]. Other studies have shown that cancer cell FAK expression and activation are linked with malignant transformation but not with an invasive phenotype in breast carcinomas [13]. Interestingly, endothelial-FAK expression in astrocytic tumours was increased in higher grade tumours [14].

Understanding the *in vivo* role of FAK has been aided by genetic ablation studies in mice. Loss of epidermal FAK can reduce tumour progression [15]. Additionally, endothelial specific FAK-kinase domain inactivation is associated with reduced vascular leakage [16]. Moreover, endothelial-FAK deletion has been shown to inhibit tumour growth due to a defect in tumour angiogenesis initiation [17]. In contrast, FAK-heterozygous mice, that have half the normal levels of FAK, display elevated xenograft tumour growth [18]. Together these results suggest that endothelial-FAK levels may affect tumour size. Despite these studies no data is available presently to link endothelial-FAK levels with prognostic factors in human breast cancer.

The increased expression of FAK in many cancer types has stimulated the development of FAK inhibitors for the treatment of cancer [19]. Given the critical role of this molecule in both the tumour and endothelial cell compartment, an analysis of the relationship between expression and clinicopathological factors would be beneficial in the design of future clinical trials targeting FAK.

The purpose of this study was to determine whether FAK expression in the endothelial cell or tumour cell compartment of invasive breast carcinomas correlates with established clinicopathological characteristics, or differences between molecular sub-types.

## Methods

### Tissue specimens

Formalin-fixed and paraffin-embedded blocks of surgically resected invasive breast cancers from 149 patients were provided by the Barts Cancer Institute Breast Tissue Bank, following informed patient consent (ethics ref:10/H0308/49). The clinicopathological characteristics (age at presentation, tumour size, tumour grade, lymph node status, and ER/PR/Her2 status) were obtained from the diagnostic histopathology reports. The tumours were allocated into molecular sub-types using the following biomarker profile: ‘luminal A’ (ER and/or PR+, Her2–), ‘luminal B’ (ER and/or PR+, Her2+), ‘Her2-positive’ (ER–, PR–, Her2+) and ‘triple negative’ (ER–, PR–, Her2–) [20]. This study followed REMARK guidelines for tumour marker prognostic studies [21].

### Immunofluorescence analysis

Sections were dewaxed in xylene and blocked in 3% H_2_O_2_ solution in methanol to block endogenous peroxidases. Antigen retrieval was performed by heating sections in 10 mM Sodium Citrate buffer. Samples were then blocked with protein block/serum free (Dako, Cambridgeshire, UK) and incubated with anti-FAK clone 4.47 (Millipore, Massachusetts, USA) and anti-PECAM antibodies (Millipore) overnight at 4˚C. Mouse and rabbit IgGs (Dako) were used as a negative control for the anti-FAK and anti-PECAM antibodies. After incubation with the primary antibodies, tissue sections were washed three times in PBS followed by 60 minutes incubation at room temperature with anti-mouse biotinylated and anti-rabbit Alexa 546 (Invitrogen Molecular Probes, Paisley, UK) antibodies. After washing with PBS, tissue sections were incubated with streptavidin-HRP for 30 minutes at room temperature (TSA/fluorescein systems; PerkinElmer, Massachusetts, USA). They were then washed with PBS and incubated for 5 minutes at room temperature with Fluorescein Tyramide solution (TSA/fluorescein systems). The sections were mounted using Prolong Gold Antifade reagent with DAPI (Invitrogen Molecular Probes, Paisley, UK). Fluorescence was analysed using the epifluorescent Zeiss Axioplan Microscope (Carl Zeiss, Germany).

### Scoring immunohistochemistry

For each case, images covering 75% to 100% of the tissue section were acquired. Each image was scored for FAK expression in tumour cells based on a scoring system that measured both percentage of positive cells (0, none; 1, <25%; 2, 25-50%; 3, 50-75%; 4, >75%) and intensity of staining (0, none; 1, weak; 2, moderate; 3, strong). The sum of these values provided a score ranging from 0–7 for each image. A mean score was then calculated for each case. In addition, each image was scored for FAK expression in tumour endothelium based on a scoring system that measured the intensity of FAK staining in each vessel (0, none; 1, borderline; 2, weak; 4, moderate; 6, strong) and took into account the percentage of positive cells per vessel. If <20% of the endothelial cells in a vessel were positive for FAK this vessel was assigned half of the intensity value of the positive cells. This system provided possible outcomes of 7 categories (0, 0.5, 1, 2, 3, 4, 6) for each blood vessel of every case. The intensity of staining was multiplied by the percentage of vessels with that score. These values were then summed to give a total score for all vessels ranging from 0 to 600. The vessels analysed had a diameter ≥5 μm and were <100 μm distance from tumour cells. The median number of vessels scored per case was 44. The samples were scored by one person in a blinded manner. To validate the score for FAK expression, 10 tumour samples were stained and scored twice on different days, providing similar scores.

### Statistical analysis

FAK expression scores for both tumour cells and blood vessels were considered as non-parametric continuous variables since neither follows a normal distribution. The correlation between FAK expression and continuous variables was performed using Spearman’s correlation co-efficient and the Mann–Whitney U test was performed to assess possible associations between FAK expression and categorical variables. The correlation between FAK expression and molecular sub-type was carried out using univariate and multivariate logistic regression with forward step-wise entry. In the regression analysis, to facilitate comparison between tumour cell and endothelial cell FAK, scores were re-scaled to give a score between 0 and 1. Scores were re-scaled by dividing the parameter score by its maximum value, so for example a raw score of 600 would be rescaled to 600/600 = 1. Alternatively, a score of 200 would be rescaled to 200/600 = 0.33. A two-sided p-value less than 0.05 was considered statistically significant. Statistical analyses were performed with SPSS statistical software, Version 19.0 (IBM Corp., Armonk, New York, USA) and GraphPad Prism, Version 4.0 (GraphPad Software Inc., La Jolla, CA, USA).

## Results

### Patient and tumour characteristics

Of the 149 patients included in this study, 129 had invasive ductal carcinoma, no special type, 15 had invasive lobular carcinoma, 2 had invasive micropapillary carcinoma and there was one patient each with mucinous, metaplastic and apocrine carcinoma. The mean age at presentation was 56.6 years and the mean tumour size was 26.6 mm. Grade 3 tumours accounted for 61% of the cohort and 48% of the patients had lymph node involvement. The luminal A sub-type was the most common, accounting for 38% of tumours. The percentage of tumours with positive ER, PR and Her2 status was 58%, 53% and 8% respectively. The clinicopathological features of the cohort are summarized in Table 1.

**Table 1:** Clinical, pathological and molecular features of the patient cohort

**Parameter**	**N**	**Mean (95% CI)/Percentage of cases (%)**
**Age**	148	56.6 years (54.5-58.6)
**Tumour size**	149	26.6 mm (23.8-29.3)
**Tumour grade**	149	
1	2	1
2	56	38
3	91	61
**Lymph node involvement**	145	
Yes	69	48
No	76	52
**ER status**	149	
Positive	87	58
Negative	62	42
**PR status**	149	
Positive	79	53
Negative	70	47
**Her2 status**	149	
Positive	12	8
Negative	137	92
**Molecular sub-type**	149	
Luminal A	56	38
Luminal B	31	21
Her2 positive	12	8
TN	50	33

Abbreviations; *CI* = confidence interval, *ER* = estrogen receptor, *PR* = progesterone receptor, *TN* = triple negative.

### Correlation between endothelial and tumour cell FAK expression

**Figure 1:**
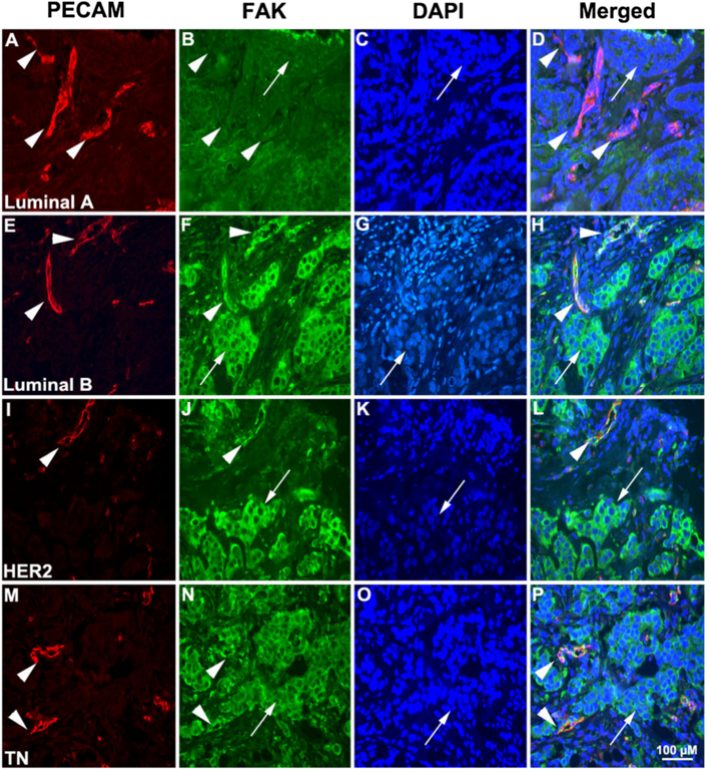
**Immunofluorescence Analysis of FAK expression in invasive breast tumour epithelium and endothelium of different ‘Intrinsic’ molecular tumour types.** Formalin fixed paraffin embedded tissue was stained for Pecam-1 (red) to identify endothelial cells and for FAK (green). Cell nuclei have been identified by counterstaining with DAPI. Characteristic examples are shown from Luminal A **(A–D)**, Luminal B **(E–H)**, Her2-overexrpessing **(I–L)** and Triple Negative **(M–P)** invasive breast tumours. All images are on x40 magnification. The analysed vessels had a diameter ≥5 μm and vessels in a ≥100 μm distance away from the tumour cell margins have been excluded. *Arrows* indicate tumour cells and *arrowheads* analysed blood vessels.

There was a positive correlation between FAK expression in the vascular and tumour cell compartments (Spearman’s correlation co-efficient = 0.394, p < 0.001). Representative immunofluorescent images of FAK expression in the tumour endothelial and cancer cell compartments in luminal A (Figure 1A–D), luminal B (Figure 1E–H), Her2-overexpressing (Figure 1I–L) and Triple Negative (Figure 1M–P) invasive breast carcinomas are shown in Figure 1. Additional file 1 shows images of tissue incubated with isotype control antibodies.

### Tumour endothelial-FAK expression and relation with clinicopathological factors

There was no significant correlation between endothelial-FAK expression and patient age at presentation or tumour size (Table 2). Significantly higher mean endothelial FAK scores were seen in grade 3 tumours (p = 0.008), ER negative tumours (p = 0.005), PR negative tumours (p = 0.002) and Her2 positive tumours (p = 0.022, Table 3). Endothelial-FAK expression was higher in TN versus non-TN tumours (p = 0.043) and lower in luminal A versus non-luminal A tumours (p < 0.001, Table 3). There were no significant differences in endothelial-FAK levels in primary tumours between lymph node positive and lymph node negative tumours, or between luminal B versus non-luminal B and Her2 positive versus non-Her2 positive sub-types, although the highest FAK scores were seen in the Her2 positive sub-type (Table 3).

**Table 2:** Correlation between FAK expression and prognostic factors (continuous variables)

**FAK location**	**Parameter**	**Correlation co-efficient**	**p-value**
Vessels	Patient age	0.096	0.246
	Tumour size	0.089	0.28
Tumour cells	Patient age	-0.138	0.094
	Tumour size	0.119	0.148

Spearman’s correlation co-efficient.

**Table 3:** Association between FAK expression and prognostic factors (categorical variables)

**FAK location**	**Parameter**	**Mean FAK score (95% CI)**	**p-value**
**Vessels**	**Tumour grade**		
	*1 or 2*	*156.2 (124.6-187.8)*	
	*3*	*200.2 (177.9-222.6)*	*0.008*
	**Lymph node involvement**		
	Yes	184.7 (157.7-211.7)	
	No	179.6 (152.8-206.5)	0.765
	**ER status**		
	*Positive*	*161.3 (139.9-184.6)*	
	*Negative*	*213.7 (184.7-242.7)*	*0.005*
	**PR status**		
	*Positive*	*156.8 (131.6-181.9)*	
	*Negative*	*212.8 (186.7-238.9)*	*0.002*
	**Her2 status**		
	*Positive*	*215.8 (181.6-250.0)*	
	*Negative*	*169.8 (148.0-191.6)*	*0.022*
	**Molecular sub-type**		
	*Luminal A vs Non-Luminal A*	*135.4 (107.6-163.2) vs 211.8 (189.0-234.6)*	*<0.001*
	Luminal B vs Non-Luminal B	208.0 (169.1-246.9) vs 176.5 (155.5-196.6)	0.130
	Her2 positive vs Non-Her2	235.9 (155.8-316) vs 178.5 (159.5-197.4)	0.121
	*TN versus Non-TN*	*208.4 (176.6-240.1) vs 170.3 (147.6-193.0)*	*0.043*
**Tumour cells**	**Tumour grade**		
	*1 or 2*	*4.1 (3.6-4.7)*	
	*3*	*5.1 (4.8-5.5)*	*0.001*
	**Lymph node involvement**		
	Yes	5.1 (4.7-5.4)	
	No	4.5 (4.0-5.0)	0.249
	**ER status**		
	*Positive*	*4.4 (4.0-4.9)*	
	*Negative*	*5.2 (4.8-5.6)*	*0.013*
	**PR status**		
	*Positive*	*4.4 (4.0-4.9)*	
	*Negative*	*5.1 (4.7-5.5)*	*0.048*
	**Her2 status**		
	Positive	5.1 (4.6-5.6)	
	Negative	4.6 (4.2-5.0)	0.215
	**Molecular sub-type**		
	*Luminal A vs Non-Luminal A*	*4.1 (3.5-4.7) vs 5.2 (4.8-5.5)*	*0.001*
	Luminal B vs Non-Luminal B	5.0 (4.4-5.6) vs 4.7 (4.3-5.0)	0.408
	Her2 positive vs Non-Her2	5.3 (4.5-6.1) vs 4.7 (4.4-5.0)	0.406
	*TN versus Non-TN*	*5.2 (4.7-5.7) vs 4.5 (4.1-4.9)*	*0.033*

Significant findings are in italics (Mann–Whitney test). Abbreviations: *CI* = confidence interval.

### FAK expression in tumour cells in relation to clinicopathological factors

There was no significant correlation between tumour cell FAK expression and patient age at presentation or tumour size (Table 2). Significantly higher mean cancer cell FAK scores were seen in grade 3 tumours (p = 0.001), ER-negative tumours (p = 0.013) and PR-negative tumours (p = 0.048, Table 3). Tumour cell FAK expression was significantly higher in TN versus non-TN tumours (p = 0.033) and lower in luminal A versus non-luminal A tumours (p = 0.001, Table 3). There was no significant difference in cancer cell FAK scores between tumours with and without lymph node involvement or between tumours with positive and negative Her2 status. As with endothelial-FAK expression, there was no significant difference between the luminal B versus non-luminal B and Her2 versus non-Her2 molecular sub-types although again, the Her2 positive sub-type had the highest absolute FAK score (Table 3).

### Correlation of FAK expression and established prognostic factors with the Luminal A sub-type – univariate regression

Given that the strongest statistical association in the sub-type analysis for both endothelial and tumour cell FAK expression was with lower scores in the luminal A versus non-luminal A tumours, we performed univariate regression analysis to identify other parameters that associated with luminal A tumours (Table 4). The 3 parameters that showed a significant association with luminal A tumours were tumour grade (p < 0.001), FAK expression in tumour cells (p = 0.001) and FAK expression in endothelial cells (p < 0.001). These three factors had an odds ratio below 1, confirming the association between low grade (grade 1/2) and low FAK expression in the luminal A sub-type (Table 4).

**Table 4:** Association between prognostic factors, FAK expression and the luminal A sub-type

**Parameter**	**LR Chi Square**	**OR (95% CI)**	**p-value**
Age at diagnosis	11.12	1.01 (0.99-1.04)	0.413
Tumour size	0.05	1.00 (0.98-1.02)	0.823
*Tumour grade*	*32.03*	*0.13 (0.06-0.27)*	*<0.001*
Lymph node status	2.06	0.61 (0.31-1.20)	0.154
*FAK tumour cells*	*11.41*	*0.11 (0.03-0.42)*	*0.001*
*FAK vessels*	*17.11*	*0.02 (0.01-014)*	*<0.001*

Significant findings are in italics (univariate logistic regression). Abbreviations: *LR* = likelihood ratio, *OR* = odds ratio, *CI* = confidence interval

### Independent correlation of endothelial-FAK with the Luminal A sub-type

To establish whether low endothelial-FAK expression was independently associated with luminal A tumours, we performed multivariate logistic regression. The parameters that associated with luminal A tumours at univariate analysis (tumour grade, endothelial-FAK expression and tumour cell FAK expression) were placed into the model in a step-wise fashion. The 2 parameters that remained in the model were tumour grade (OR 0.14, 95% CI: 0.07-0.31; p < 0.001) and endothelial-FAK expression (OR 0.03, 95% CI: 0.01-0.25; p = 0.001, Table 5), suggesting that low endothelial-FAK expression is independently associated with the luminal A sub-type, even after taking tumour grade into account.

**Table 5:** Multivariate model containing parameters predictive of the luminal A sub-type

**Parameter**	**Stepwise entry**	**LR Chi Square**	**OR (95% CI)**	**p-value**
Tumour Grade	1	32.03	0.14 (0.07-0.31)	<0.001
FAK vessels	2	11.31	0.03 (0.01-0.25)	0.001

Multivariate logistic regression with stepwise forward entry. Abbreviations: LR = likelihood ratio, OR = odds ratio, CI = confidence interval.

## Discussion

The aim of the current study was to determine whether the levels of endothelial and tumour cell FAK correlate with clinicopathological characteristics in invasive breast carcinoma. While low expression of both endothelial and tumour cell FAK associated with luminal A tumours, only endothelial-FAK was independently associated with these tumours in multivariate analysis. This is the first study to demonstrate a relationship between endothelial-FAK expression and molecular sub-type in invasive breast cancer and our findings suggest that vascular expression of FAK is potentially more clinically relevant than tumour cell FAK in breast cancer.

The importance of FAK in angiogenesis and in cancer progression has been shown in several animal studies [15,17,18,22-24]. These studies in combination with the observed upregulation of FAK in several epithelial cancers has initiated the development of FAK inhibitors for the treatment of cancer [19].

Previous studies have investigated the significance of tumour cell FAK expression in invasive breast cancer [12,25-28]. Few of these studies looked specifically at molecular sub-type, but Yom et al. found that low tumour cell FAK expression correlated with the luminal A sub-type and higher levels with the luminal B and TN sub-types at univariate analysis [28] and our results corroborate these findings. In particular our finding of increased tumour cell and endothelial cell FAK in TN tumours suggest that FAK likely plays a role in the biology of these tumours. Predictably, our results regarding molecular sub-type are mirrored by our observations of the individual steroid receptors and Her2, where increased FAK expression correlated with ER and PR negativity and Her2 positivity. Others have shown comparable findings [12,25,28]. Interestingly, in a study investigating the relationship between FAK and major signaling pathways in 162 node-negative breast cancers, elevated FAK expression correlated with Her2 over-expression and phospho-Src Tyr-215, prompting the authors to speculate that the activation of Akt via the FAK pathway contributes to the aggressive nature of Her2 over-expressing tumours [12]. Although we didn’t find a statistically significant increase in endothelial/tumour cell FAK in the Her2 positive/luminal B sub-types (versus non-Her2 positive/non-luminal B tumours) the absolute scores were higher in the former and the lack of significance may reflect the smaller patient numbers in these groups.

We found higher FAK expression (endothelial and tumour cell) in more aggressive grade 3 tumours, compared to grades 1 and 2. This is in keeping with other studies that have evaluated tumour cell FAK expression in tissue and cytology specimens from invasive breast cancers [12,25,27,28]. Tumour grade is an established poor prognostic factor in breast cancer [29] and given the association between high grade (and other prognostic factors such as ER/PR negativity) and high FAK expression it is entirely possible that FAK over-expression is associated with a poor outcome. To date, studies of outcome in relation to protein expression of FAK in human breast cancers have not demonstrated a significant effect on survival [12,28], and larger studies with long term follow-up are needed. Of note, FAK amplification/high polysomy has been shown to be an independent poor prognostic factor for both overall and relapse-free survival [28].

A previous clinical trial looking at the VEGFR inhibitor, Sunitinib in unselected breast cancer patients has been unsuccessful [30]. Given the association between increased VEGF-receptor 2 expression in TN breast cancer [31], targeting TN breast cancer with Sunitinib or the anti-VEGF agent Bevacizumab may be a more effective approach and these clinical trials are now ongoing [30,32]. Likewise, our results suggest that clinical trials should consider focusing on non-luminal A tumours in the evaluation of FAK inhibitors for the treatment of breast cancer. Moreover, since the expression of FAK is not limited to a single cancer compartment effective inhibition of FAK signaling is particularly appealing.

## Conclusion

In conclusion, this study is the first to analyse endothelial-associated FAK expression in human breast tumour samples. We demonstrate that lower endothelial FAK expression is independently associated with the luminal A sub-type, and conversely, high endothelial and tumour cell FAK expression correlates with the poorer prognosis non-luminal A tumours and other established poor prognostic factors. The association between high FAK levels and TN tumours is worthy of further investigation in a larger series to establish the prognostic significance of tumour/endothelial FAK in the TN/BL sub-type. Overall our findings strengthen the argument for investigating the role of FAK inhibitors as a novel treatment for poor prognosis breast cancer sub-types and identify endothelial expression of the protein as a potentially useful biomarker for future clinical studies.

## Competing interests

The authors declare that they have no competing interests.

## Authors’ contributions

ANA, VP and KHD designed the experiments and the paper. JLJ provided human tissue sections. CMH carried out the statistical analysis and comparisons. GE cut sections for analysis. ANA, CMH, JLJ and KHD wrote the paper. All authors read and approved the final manuscript.

## Pre-publication history

The pre-publication history for this paper can be accessed here:

http://www.biomedcentral.com/1471-2407/14/237/prepub

## Supplementary Material

Additional file 1 **Negative controls for immunofluorescence staining.** Formalin fixed paraffin embedded IDC tissue was incubated with rabbit IgG and mouse IgG antibodies, followed by anti-rabbit (Alexa 546; red) and anti-mouse (Alexa-488; green) secondary antibodies. Cell nuclei have been identified by counterstaining with DAPI. Click here for file
