# Supplementary figures and images for: Tumour-associated endothelial-FAK correlated with molecular sub-type and prognostic factors in invasive breast cancer
Source: BMC Cancer. 2014 Apr 2;14:237. doi: 10.1186/1471-2407-14-237 (PMC3997837; doi:10.1186/1471-2407-14-237)

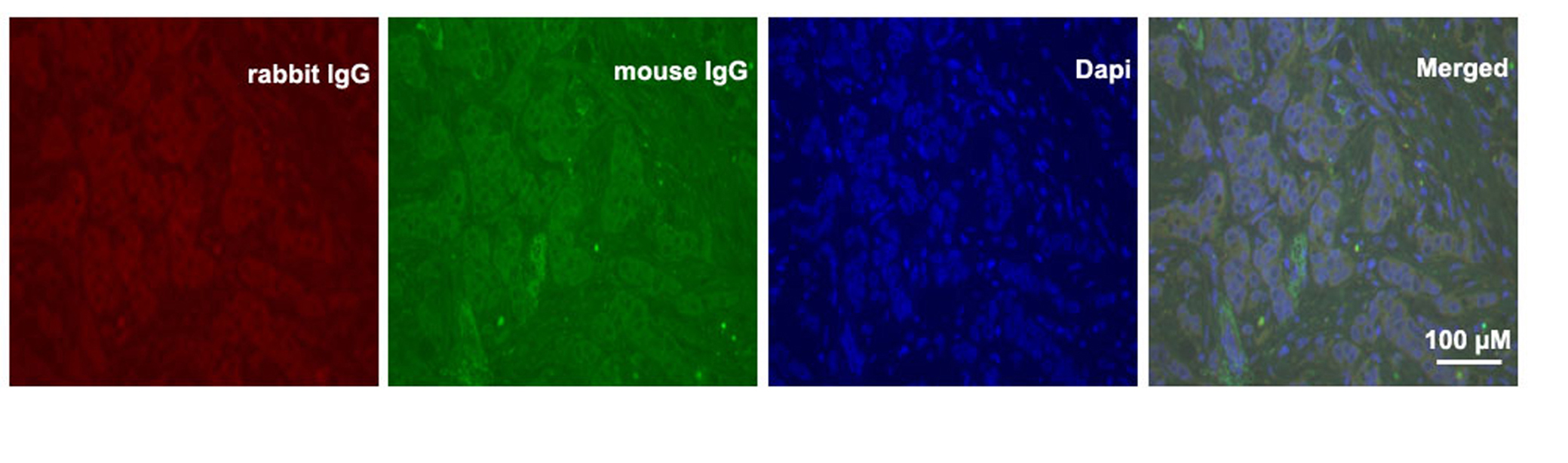

Supplement: Additional file 1 — Negative controls for immunofluorescence staining. Formalin fixed paraffin embedded IDC tissue was incubated with rabbit IgG and mouse IgG antibodies, followed by anti-rabbit (Alexa 546; red) and anti-mouse (Alexa-488; green) secondary antibodies. Cell nuclei have been identified by counterstaining with DAPI. [file 1471-2407-14-237-S1.jpeg]
